# Bacterial etiology and risk factors among newborns suspected of sepsis at Hawassa, Ethiopia

**DOI:** 10.1038/s41598-022-24572-0

**Published:** 2022-11-23

**Authors:** Endale Worku, Demissie Assegu Fenta, Musa Mohammed Ali

**Affiliations:** 1 Hawassa University Comprehensive Specialized Hospital, Hawassa, Ethiopia; 2 School of Medical Laboratory Science, College of Medicine and Health Sciences, Hawassa University, Hawssa, Ethiopia

**Keywords:** Microbiology, Diseases

## Abstract

Neonatal sepsis is a systemic infection that occurs at an early age. Its etiology varies from one region to the other. The contribution of sepsis to neonatal mortality and morbidity is significant in resource-limited countries; however, there is limited information about the etiology of sepsis in Sidama Regional State, Ethiopia. The aim of this study was to determine the prevalence of bacterial caused newborn sepsis, associated factors, and the antimicrobial susceptibility profile of bacteria. A hospital-based prospective cross-sectional study was conducted among 392 sepsis suspected newborns admitted to the neonatal intensive care unit of Hawassa University Comprehensive Specialized Hospital from March 2021 to November 2021. Blood specimens were collected and bacteria were isolated using the standard culture method. The drug resistance profile of bacteria was evaluated using the disk diffusion method. The socio-demographic and clinical parameters of participants were gathered using a questionnaire. Binary logistic regression was used to determine the determinants of sepsis. A variable with a *p* < 0.05 was considered a significant determinant of neonatal sepsis with a 95% confidence level. The prevalence of sepsis caused by bacteria among newborns was 143 (36.5%); 95% CI (31.3–41.4). The predominant bacteria was *Klebsiella* species (n = 61; 42.65%), followed by non-lactose fermenting Gram-negative bacteria (n = 27; 18.88%) and *Enterococcus* species (n = 26; 18.18%). The overall proportions of antimicrobial resistance of Gram-negative bacteria range from 10.2 to 99.1%. All *Klebsiella* species were resistant to ceftriaxone. Ppremature rupture of membrane [AOR = 12.7 (95% CI 6.430–25.106)], absence of respiratory support [AOR = 3.53 (95% CI 1.840–6.759)], sex of newborns [AOR = 2.10 (1.214–3.560)] and reason for admission [AOR = 3.17 (95% CI 1.278–7.859)] were significantly associated with culture-confirmed neonatal sepsis. This study indicated the contribution of bacteria in causing sepsis among newborns; the majority of them were Gram-negative bacteria. Most recovered bacteria were resistant to commonly used antibiotics. Pre-term, mode of delivery and types of respiratory support were significantly associated with the occurrence of sepsis caused by bacteria.

## Introduction

Neonatal sepsis is a systemic infection that occurs during the first ages of life^1^. It is a major cause of morbidity and mortality worldwide accounting for about 26%^2^. Neonatal sepsis is broadly divided into two types according to the age of onset: Early-onset sepsis if signs and symptoms of sepsis appeared within the first seven days of life and classified as late-onset sepsis if clinical features of sepsis are presented between 7 and 90 days of age^3^.

In developing countries, 30–50% of neonatal mortality is attributed to sepsis. In spite of recent advances in health care, late identification and inappropriate treatment remain crucial factors causing high neonatal mortality^4^. The neonatal period is the most vulnerable time for children’s survival because of the immature immune system. Globally, every year about 4 million children die in the first 4 weeks of life, of which 99% of the deaths occur in low- and middle-income countries^1^. Factors such as asphyxia, low birth weight, prematurity, delivery settings, mode of delivery, newborn mixed feeding, and antenatal care received are believed to contribute to the incidence of neonatal sepsis across^5^.

Neonatal sepsis could be of bacterial, viral, fungal, or rickettsia origin^6^. Over 40% of under-five deaths occur worldwide among newborns, leading to 3.1 million neonatal deaths annually^7^. Generally, the mother's colonized genitourinary tract is the source of infection for EOS; the common etiologies are Group B *Streptococci*, *Escherichia coli,* Coagulase-negative *Staphylococci*, *Haemophilus influenza,* and *Listeria monocytogenes*. The etiologies of LOS are horizontally acquired and include Coagulase-negative *Staphylococci, Staphylococcus aureus, E. coli, Klebsiella* species, *Pseudomonas* species, *Candida* species, *Acinetobacter* species, and anaerobes^7^. The mechanism of transmission is unclear where different environmental risk factors may affect paths of transmission. LOS is common among newborns with low-birth-weight infants and who underwent invasive procedures^2^.

The spectrum of presentation of neonatal sepsis is varied and includes hypothermia, fever, lethargy, poor cry, refusal to suck or poor feeding, off-color, poor perfusion, and shock, absent neonatal reflexes, bradycardia/tachycardia, respiratory distress, hypo/hyperglycemia, and metabolic acidosis. The etiology of septicemia is multifactorial. Neonatal sepsis is caused by a variety of Gram-positive as well as Gram-negative bacteria, and sometimes yeasts^8^. The types of organisms that cause neonatal sepsis change over time and vary from region to region^9^. Blood culture remains the gold standard for diagnosis of neonatal sepsis, regardless of its low sensitivity which may be due to the small volume of the blood sample or the use of empirical antibiotics prior to sampling^10^. In this study, we envisaged determining the etiology and associated factors of sepsis among clinically suspected newborns.

## Methods

### Study design

A hospital-based cross-sectional prospective study was conducted from March 2021 to November 2021 at Hawassa University Comprehensive Specialized Hospital (HUCSH) Neonatal Intensive Care Unit (NICU). HUCSH is located in Hawassa city, Sidama Regional State, Ethiopia. HUCSH provides healthcare-related services for over 18 million populations.

### Study population

All newborns who were admitted to the NICU of HUCSH and with signs and symptoms of sepsis were considered as the study population. Newborns with the following conditions were included in the study: age < 90 days, signs and symptoms of sepsis which include hypothermia, fever, lethargy, poor cry, refusal to suck or poor feeding, poor perfusion, and shock, absence of neonatal reflexes, bradycardia or tachycardia, respiratory distress, hypo or hyperglycemia, and metabolic acidosis. Newborns on antibiotics during birth and/or in the last ten days were excluded from the study. Newborns without family members that can provide background information and congenital anomalies were also excluded.

The sample size was determined using a single population proportion formula by taking the prevalence of neonatal sepsis (36.6%) reported from Tigray, Northern Ethiopia^11^, the margin of error (5%), and 95% Confidence level (z = 1.96). After considering 10% for the non-response rate the sample size was 392. A consecutive convenient sampling technique was used to recruit study participants until the sample size was reached.

### Socio-demographic and clinical data collection

The socio-demographic and clinical data were collected using a pretested structured interviewer-administered questionnaire. Before data collection, the data collector explained the purpose of the study, the objectives of the study, the procedure to be carried out, and informed consent obtained from parents/guardians of newborns.

### Culture and identification of bacteria

Before blood sample collection, the site was cleaned with 70% isopropyl alcohol and 2% tincture of iodine twice. Two milliliters of venous blood were aseptically collected from the antecubital fossa or femoral vein of sepsis suspected newborns using a butterfly needle by an attending nurse and dispensed aseptically into a sterilized universal bottle containing 20 ml of tryptone soy broth to make a 1:10 dilution. Before transferring the sample to the bottle, the top of the blood culture bottle was cleansed with an ethanol-ether swab and the needle was inserted through the rubber liner and blood was injected gently. Inoculated bottles were transported to the laboratory within 2 h.

The inoculated blood culture bottles were incubated aerobically at 35–37 °C for 7 days and examined daily for signs of bacterial growth. For a bottle that shows signs of bacterial growth, a sample was subcultured onto blood agar, chocolate agar, MacConkey agar, and mannitol salt agar. Inoculated plates were incubated at 35–37 °C for 24 h in appropriate environmental conditions. Blood culture bottles without signs of bacterial growth after seven days were also subcultured. If there is no growth after the subculture it was regarded as negative. Bacteria were identified based on colony appearance, Gram reaction, and biochemical tests.

### Antibacterial susceptibility testing

Antibacterial susceptibility testing was performed using the disc diffusion method according to the criteria of the Clinical and Laboratory Standards Institute guidelines. Briefly, from a pure culture, 3–5 selected colonies of bacteria were transferred into a tube containing 5 ml sterile normal saline and mixed gently until a homogenous suspension that matches McFarland 0.5 turbidity was attained. A sterile cotton swab was used to uniformly inoculate a suspension of bacteria on the Mueller Hinton agar. The plates were placed at room temperature for 3‑5 min and then antibiotic discs were applied on the surface of the inoculated Mueller–Hinton plate. The inoculated plates were incubated at 37 °C for 18–24 h. Tested antibiotics include ampicillin (10 μg), vancomycin (30 μg), ciprofloxacin (5 μg), gentamycin (10 μg), amikacin (30 μg), penicillin (10units), amoxicillin-clavulanate (20/10 µg), chloramphenicol (30 µg), ceftriaxone (30 µg), cefotaxime (30 µg), clindamycin (2 µg), cefepime (5 µg), erythromycin (15 µg), meropenem (10 µg), imipenem (10 µg), and piperacillin/tazobactam (100/10 µg).

## Quality control

Before data collection, questionnaires were translated from English to Amharic and then translated back to English by another person to check for their consistency. Prior to actual data collection, the quality of data was assured by pre-testing of questionnaires among a population representing 5% of the sample size. Each batch of the prepared media was checked for their sterility by incubating 5% of the culture media at 37 °C for 24 h. To check the performance of culture media and antibiotics we used reference strains such as *E. coli* (ATCC-25922), *S. aureus* (ATCC-25923), and *P. aeruginosa* (ATCC-27853).

### Data analysis

All questionnaires were checked visually, coded, and entered into SPSS version 25.0 for analysis. For controlling errors, the questionnaires were double entered; and also frequency check was performed. To describe the study population in relation to relevant variables, descriptive statistics (frequencies, mean, standard deviation, and percentage) were used. Binary logistic regression was used to assess the association between outcome and explanatory variables. Associations between dependent and independent variables were assessed and their strength was described using 95% confidence intervals. Bivariable binary logistic regression was carried out then variables with *p* < 0.25 were further analyzed by multivariable binary logistic regressions. *p* < 0.05 was accepted as statistically significant, and the results were presented using text, tables, charts, and graphs.

### Ethical consideration

The study was approved by the institutional review board (IRB) of Hawassa University College of medicine and health science (Reference No.: IRB/092/13). Before enrolling eligible study participants; the purpose, objective, benefits, and risks of the study were explained to parents/guardians. The data were collected after written informed consent was obtained from a parent or guardian. All methods were performed in accordance with the relevant guidelines and regulations mentioned by the Declaration of Helsinki.

### Operational definition

Suspected neonatal sepsis: Neonates presented with any one of the systemic manifestations of danger signs: not feeding well, convulsions, drowsy or unconscious, movement only when stimulated or no movement at all, fast breathing (60 breaths per min), grunting severe chest in-drawing, raised temperature > 38 °C, hypothermia < 35.5 °C, central cyanosis or could be severe jaundice, severe abdominal distension or localizing signs of infection were diagnosed as having neonatal sepsis.

*Early-onset of sepsis* If sepsis occurs in newborns within period of 0 to 7 days after birth.

*Late-onset of sepsis* If sepsis occurs in newborns between after 7 to 90 days of life.

*Perinatal asphyxia* is an oxygen deficient state from the 28th week of gestation to the first seven days following delivery.

*Premature rupture of membranes* (PROM) is a rupture of the amniotic sac before labour begins.

*Respiratory support* refers to the various medical procedures and equipment used to treat pulmonary diseases.

## Results

### Socio-demographic-characteristics

A total of 392 newborns suspected of sepsis were included in this study with an overall response rate of 100%. The median age of the mothers was 30 (16–43) years. The place of residence for 259 (66.1%) mothers was urban. Most mothers completed primary school (Table 1).

**Table 1:** Socio-demographic characteristics of mothers of newborns attending neonatal intensive care unit of Hawassa University Comprehensive Specialized Hospital, Hawassa, Ethiopia, March 2021–November 2021 (N = 392).

Variables	Category	Frequency (%)
Age of mothers in year	15–19	6 (1.5)
	20–24	25 (6.4)
	25–29	129 (32.9)
	30–34	207 (52.8)
	> 35	25 (6.4)
Place of residence	Urban	259 (66.1)
	Rural	133 (33.9)
Educational level	No formal education	65 (16.6)
	Primary (1–8 Grade)	155 (39.5)
	Junior secondary school (9–10 Grade)	74 (18.9)
	Higher secondary school (11–12 Grade )	54 (13.8)
	Collage level	44 (11.2)
Monthly income of the house hold in ethiopian Birr	≤ 2500.00	52 (13.3)
	> 2500.00	340 (86.7)
Parity	Premipara	97 (24.7)
	Multipara	295 (75.3)
Length of admission during Post-delivery in hours	< 6 h	143 (36.5)
	6–12	203 (51.8)
	13–24	26 (6.6)
	25–72	9 (2.3)
	> 72 h	11 (2.8)

### Characteristics of newborns

Of the total newborns who participated in this study, 223 (56.9%) were males whereas 169 (43.1%) were females. the median age of the newborns was 4 (1–90) days. The majority of patients were aged less than or equal to seven days (58.9%). Of the total newborns, 96 (24.5%) were preterm; the mode of delivery for 246 (62.8%) newborns was vaginal. 173 (44.1%) participants had low birth weight (< 2500 g) and for 350 (89.3%) place of delivery was Hospital (Table 2).

**Table 2:** Characteristics of newborns suspected of sepsis attending neonatal intensive care unit of Hawassa University Comprehensive Specialized Hospital, March 2021–November 2021 (N = 392).

Variables	Category	Frequency (%)
Sex	Male	223 (56.9)
	Female	169 (43.1)
Weight in grams	< 2500	173 (44.1)
	≥ 2500	211 (53.9)
	Unknown	8 (2.0)
Place of birth	Home	6 (1.5)
	Health center	37(9.5)
	Hospital	349 (89.0)
Types of suspected sepsis	Early onset sepsis	231 (58.9)
	Late onset sepsis	161 (41.1)
Gestational age	Preterm (≤ 36wks)	96 (24.5)
	Full-Term (37–40wks)	296 (75.5)
Length of hospital stay in days	≤ 7	231 (58.9%)
	≥ 8	161 (41.1)
Mode of delivery	Spontaneous vaginal delivery	246 (62.8)
	Instrumental vaginal delivery	64 (16.3)
	Caesarean section	82 (20.9)

### Prevalence of culture-confirmed sepsis

Out of the total of 392 newborns suspected of sepsis, 143 were blood culture-positive with a prevalence of 36.5% (95% CI 31.3–41.4). The predominant bacteria were *Klebsiella* species (n = 61; 42.65%), followed by, non-lactose fermenting Gram-negative bacteria (n = 27; 18.88%) and *Enterococcus* species (n = 26; 18.18%). All infections were due to single bacteria. Gram-negative and Gram-positive bacteria constituted (n = 108; 75.5%) and (n = 35; 24.5%) respectively. Among the culture-proven cases, 76 (53.1%) occurred in newborns aged ≤ 7 days. The predominant bacteria during the first week of life were Gram-negative bacteria, accounting for 66 (86.8%). Among newborns within 8 and 90 days of life, Gram-negative and Gram-positive bacteria accounted for 42 (62.7%) and 25 (37.3%) respectively (Table 3).

**Table 3:** Distribution of bacteria across early-onset and late-onset sepsis at neonatal intensive care unit of Hawassa University Comprehensive Specialized Hospital, Hawassa, Ethiopia, March 2021–November 2021 (N = 143).

Bacterial isolates	Types of sepsis		
	Early-onset n (%)	Late-onset n (%)	Total n (%)
*Klebsiella* species* (n = 61)	36 (47.4)	25 (43.7)	61 (42.7)
*Enterococcus* species (n = 26)	3 (3.9)	19 (28.4)	22 (15.4)
*Acinetobacter* species (n = 16)	10 (13.2)	6 (9)	16 (11.2)
*Pseudomonas* species (n = 11)	5 (6.6)	6 (9)	11 (7.7)
*Staphylococcus aureus* (n = 9)	3 (3.9)	6 (9)	9 (6.2)
*Escherichia coli* (n = 7)	4 (5.3)	3 (4.5)	7 (4.9)
*Enterobacter aggoelmerans* (6)	6 (7.9)	–	6 (4.2)
*Enterobacter* species (4)	2 (2.6)	2 (3)	4 (2.8)
*Seratia marcencences* (2)	2 (2.6)	–	2 (1.4)
*Citrobacter* species (n = 1)	1 (1.3)	–	1 (0.7)
Total (n = 143)	76 (100)	67 (100)	143 (100)

*Klebsiella pneumoniae = *46, Klebsiella ozenae* = *13, Klebsiella oxytoca* = *2.*

### Factors associated with culture-confirmed sepsis

In a bivariable analysis model, nine variables such as place of delivery, gestational age, premature rupture of membrane, mode of delivery, age of newborns, sex of newborns, APGAR score in the 5th minute, the reason for admission to NICU, and respiratory support had a *p* < 0.25. As they fulfilled the screening criteria, they were analyzed using multivariable binary logistic regression. In multivariable analysis, premature rupture of membrane [AOR = 12.7 (95% CI 6.430–25.106)], absence of respiratory support [AOR = 3.53 (95% CI 1.840–6.759)], mode of delivery; instrumental vaginal delivery [AOR = 0.38 (95% CI (0.192–0.782)] and (Caesarean section) [AOR = 0.42 (95% CI 0.217–0.803)], sex of newborns (female) [AOR = 2.10 (1.214–3.560)] and reason for admission to NICU such as, low birth weight [AOR = 3.17 (95% CI 1.278–7.859)] were significantly associated with culture-confirmed neonatal sepsis (Table 4).

**Table 4:** Analysis of factors associated with culture-confirmed sepsis among newborns at Hawassa University Comprehensive Specialized Hospital, Hawassa, Ethiopia February 2021 to November 2021.

Variables	Culture result		COR (95% CI)	*p* value	AOR (95% CI)	*p* value
	Positive (n = 143) (%)	Negative (n = 249) (%)				
**Place of delivery**						
Home	1 (16.7)	5 (83.3)	2.76 (0.318–23.851)	0.351	1.47 (0.132–16.239)	0.757
health center	18 (48.7)	19 (51.3)	0.58 (0.294–1.149)	0.119	0.45 (0.184–1.091)	0.077
Hospital	124 (35.5)	225 (64.5)	1		1	
**Gestational age**						
Preterm	41 (42.7)	55 (57.3)	1.42 (0.886–2.269)	0.146	0.79 (0.425–1.458)	0.446
Full term	102 (34.5)	194 (65.5)	1		1	
**Premature rupture of membrane**						
Yes	63 (77.8)	18 (22.2)	10.10 (5.646–18.091)	< 0.001	12.70 (6.430–25.106)	< 0.001
No	80 (25.7)	231 (74.3)	1		1	
**Way of delivery**						
Spontaneous Virginal delivery	68 (27.6)	178 (72.4)	1		1	
Instrumental vaginal delivery	32 (50)	32 (50)	0.38 (0.217–0.672)	0.001	0.38 (0.192–0.782)	0.008
Cesarean section	43 (52.4)	39 (47.6)	0.35 (0.207–0.580)	< 0.001	0.42 (0.217–0.803)	0.009
**Sex of newborns**						
Male	88 (39.5)	135 (60.5)	1		1	
Female	55 (32.5)	114 (67.5)	1.35 (0.889–2.055)	0.159	2.10 (1.214–3.560)	0.008
**Age of newborns in day**						
≤ 7	76 (32.9)	155 (67.1)	1		1	
≥ 8	67 (41.6)	94 (58.4)	0.72 (0.476–1.092)	0.122*	0.81 (0.464–1.428)	0.473
**APGAR score in5th min**						
Low (< 7)	43 (50.6)	42 (49.4)	1		1	
Normal ( ≥)	100 (32.6)	207 (67.4)	2.12 (1.301–3.451)	0.003*	1.24 (0.659–2.337)	0.504
**Reason for admission to NICU**						
LBW	30 (25.6)	87 (74.4)	3.63 (1.897–6.926)	< 0.001	3.17 (1.278–7.859)	0.013
Hypothermia	10 (27)	27 (73)	3.38 (1.401–8.132)	0.007	1.88 (0.619–5.681)	0.266
Sepsis	29 (31.5)	63 (68.5)	2.72 (1.398–5.273)	0.003	1.96 (0.839–4.565)	0.120
Respiratory distress	39 (47)	44 (53)	1.41 (0.730–2.723)	0.306	2.76 (1.234–6.158)	0.130
Prenatal asphyxia	35 (55.6)	28 (44.4)	1		1	
**Respiratory support**						
Absence of respiratory support	38 (21.1)	142 (78.9)	3.95 (2.490–6.256)	< 0.001	3.53 (1.840–6.759)	< 0.001
Oxygen mask	11 (37.9)	18 (62.1)	1.73 (0.773–3.862)	0.182	2.63 (0.984–7.008)	0.054
Nasal prong	94 (51.4)	89 (48.6)	1		1	

*COR* crude odds ratio, *AOR*adjusted odds ratio, *CI* confidence interval.

### Antimicrobial resistance profile

The overall antimicrobial resistance level for Gram-negative bacteria ranges from 10.2 to 99.1%. All *Klebsiella* species were resistant to ceftriaxone while most showed susceptibility to imipenem (93.5%), meropenem (91.8%), and ciprofloxacin (86.9%). 85.8% of *E. coli* was resistant to sulfamethoxazole-trimethoprim, cefepime, and ceftriaxone (Table 5).

**Table 5:** Antibiotic Susceptibility Patterns of Gram-Negative Bacteria isolated from newborn suspected of sepsis at Hawassa University Comprehensive Specialized Hospital February 2021 to November 2021 (n = 108).

Bacteria	Susceptibility profile of n (%)													
	AMK	CEP	CRO	CAZ	CIP	CHL	AMC	IMP	MER	CN	SXT	TOB	PIP/TAZ	
*Klebsiella* species* (n = 61)	R	18 (29.5)	61 (100)	59 (96.7)	61 (100)	5 (8.2)	12 (19.7)	60 (98.4)	4 (6.5)	5 (8.2)	22 (36.1)	57 (93.5)	ND	ND
	I	–	–	–	–	3 (4.9)	–	1 (1.6)	–	–	1 (1.6)	–	ND	ND
	S	43 (70.5)	–	2 (3.3)	–	53 (86.9)	49 (80.3)	–	57 (93.5)	56 (91.8)	38 (62.3)	4 (6.5)	ND	ND
*Acinetobacter* species (n = 16)	R	6 (37.5)	16 (100)	14 (87.5)	15 (93.7)	5 (31.3)	10 (62.5)	16 (100)	6 (37.5)	5 (31.3)	5 (31.3)	11 (68.7)	4 (25)	3 (18.8)
	I	–	–	1 (6.3)	1 (6.3)	2 (12.5)	1 (6.3)	–	1 (6.3)	2 (12.5)	1 (6.25)	1 (6.3)	1 (6.3)	1 (6.3)
	S	10 (62.5)	–	1 (6.3)	–	9 (56.3)	5 (31.3)	–	9 (56.3)	9 (56.3)	10 (62.5)	4 (25)	11 (68.7)	12 (75)
*Pseudomonas* species (n = 11)	R	5 (45.5)	10 (90.9)	9 (81.8)	11 (100)	4 (36.4)	5 (45.5)	11 (100)	1 (9.1)	1 (9.1)	6 (54.5)	6 (54.5)	2 (18.2)	1 (9.1)
	I	–	–	–	–	1 (9.1)	1 (9.1)	–	1 (9.1)	1 (9.1)	1 (9.1)	–	–	–
	S	6 (54.5)	1 (9.1)	2 (18.2)	–	6 (54.5)	5 (45.5)	–	9 (81.8)	9 (81.8)	4 (36.4)	5 (45.5)	9 (81.8)	10 (90.9)
*Escherichia coli* (n = 7)	R	2 (28.6)	6 (85.7)	6 (85.7)	7 (100)	3 (42.8)	3 (42.9)	7 (100)	–	–	3 (42.9)	6 (85.7)	ND	ND
	I	1 (14.3)	–	–	–	–	–	–	–	–	1 (14.2)	–	ND	ND
	S	4 (57.1)	1 (14.3)	1 (14.3)	–	4 (57.1)	4 (57.2)	–	7 (100)	7 (100)	3 (42.9)	1 (14.3)	ND	ND
*Citrobacter* species (n = 1)	R	–	1 (100)	1 (100)	1 (100)	1 (100)	1 (100)	1 (100)	–	–	1 (100)	1 (100)	ND	ND
	I	–	–	–	–	–	–	–	–	–	–	–	ND	ND
	S	1 (100)	–	–	–	–	–	–	1 (100)	1 (100)	–	–	ND	ND
*Serratia marcescens* (n = 2)	R	2 (100)	2 (100)	2 (100)	2 (100)	–	2 (100)	2 (100)	–	–	2 (100)	–	ND	ND
	I	–	–	–	–	–	–	–	–	–	–	–	ND	ND
	S	–	–	–	–	2 (100)	–	–	2 (100)	2 (100)	–	2 (100)	ND	ND
*Enterobacter aglemerans* (n = 6)	R	–	5 (83.3)	6 (100)	6 (100)	–	1 (16.7)	6 (100)	–	–	5 (83.3)	3 (50)	ND	ND
	I	–	–	–	–	–	–	–	–	–	–	–	ND	ND
	S	6 (100)	1 (16.7)	–	–	6 (100)	5 (83.3)	–	6 (100)	6 (100)	1 (16.7)	3 (50)	ND	ND
*Enterobacter* Species (n = 4)	R	1 (25)	4 (100)	4 (100)	4 (100)	–	1 (25)	4 (100)	–	–	1 (25)	3 (75)	ND	ND
	I	–	–	–	–	–	–	–	–	–	–	–	ND	ND
	S	3 (75)	–	–	–	4 (100)	3 (75)	–	4 (100)	4 (100)	3 (75)	1 (25)	ND	ND
Total (n = 108)	R	34 (31.5)	105 (97.2)	101 (93.5)	107 (99.1)	18 (16.67)	35 (32.40)	107 (99.1)	11 (10.2)	11 (10.2)	45 (41.67)	87 (80.56)	6 (22.2)	4 (14.8)
	I	1 (0.9)	–	1 (0.92)	1 (0.9)	6 (5.56)	2 (1.8)	1 (0.9)	2 (1.8)	3 (2.78)	4 (3.7)	1 (0.9)	1 (3.7)	1 (3.7)
	S	73 (67.6)	3 (2.8)	6 (5.6)	–	84 (77.8)	71 (65.7)	–	95 (87.9)	94 (87.0)	59 (54.6)	20 (18.5)	20 (74.0)	22 (81.5)

*AMK* Amikacin, *CEP* Cefepime, *CN* Gentamicin, *CAZ* Ceftazidime, *AMC* Amoxicillin-Clavulanate, *CHL* Chloramphenicol; *CRO* Ceftriaxone; *CIP* Ciprofloxacin; *SXT* Sulfamethoxazole-Trimethoprim; *IMP* Imipenem, *MRO* Meropenem, *PRL/TAZ* Piperacillin-Tazobactam, *TOB* Tobramycin *R* Resistant, *I* Intermediate, *S* Susceptible, *ND* Not determined.

** *Klebsiella pneumoniae* = *46, Klebsiella ozenae* = *13, Klebsiella oxytoca* = *2.*

Of the total Gram-positive bacteria, 82.9%, 80%, 55.6%, 44.5%, 33.3%, and 30.8% were resistant to penicillin, ampicillin, sulfamethoxazole-trimethoprim, gentamicin, clindamycin, and vancomycin respectively (Table 6).

**Table 6:** Antibiotic susceptibility profile of Gram-positive bacteria isolated from newborn suspected of sepsis at Hawassa University Comprehensive specialized Hospital February 2021 to November 2021 (n = 35).

Bacteria	Susceptibility profile n (%)											
	AMP	PEN	CN	E	CHL	VAN	OX (FOX)	LIN	SXT	CLN	CIP	
*Enterococcus* Species (26)	R	26 (100)	24 (92.3)	ND	ND	2 (7.7)	8 (30.7)	ND	ND	ND	ND	ND
	I	–	–	ND	ND	3 (11.5)	–	ND	ND	ND	ND	ND
	S	–	2 (7.7)	ND	ND	21 (80.7)	18 (69.3)	ND	ND	ND	ND	ND
*Staphylococcus aureus* (9)	R	2 (22.2)	5 (55.6)	4 (44.4)	1 (11.1)	ND	ND	2 (22.2)	–	5 (55.6)	3 (33.3)	–
	I	–	1 (11.1)	1 (11.1)	–	ND	ND	–	–	3 (33.3)	–	–
	S	7 (77.77)	3 (33.3)	4 (44.4)	8 (88.9)	ND	ND	7 (77.8)	9 (100)	1 (11.1)	6 (66.7)	9 (100)
Total (35)	R	28 (80)	29 (82.9)	4 (44.5)	1 (11.1)	2 (7.7)	8 (30.8)	2 (22.2)	–	5 (55.6)	3 (33.3	–
	I	–	1 (2.9)	1 (11.1)	–	3 (11.5)	–	–	–	3 (33.3)	–	–
	S	7 (20)	5 (14.2)	4 (44.5)	8 (88.9)	21 (80.8)	18 (69.2)	7 (77.8)	9 (100)	1 (11.1)	6 (66.7)	9 (100)

*AMP* Ampicillin, *P* Penicillin, *CN* Gentamicin, *E* Erythromycin, *CLN* Clindamycin, *SXT* sulfamethoxazole-trimethoprim, *CIP* Ciprofloxacin, *OX* Oxacillin, *VAN* Vancomycin, *CHL* Chloramphenicol, *IN* Linezolid, *R* Resistance, *I* Intermediate, *S* Susceptible, *ND* not determined.

### Multidrug resistance profile

Of the total of 143 isolates tested for antimicrobial susceptibility, multi-drug resistance (resistance ≥ drug categories) was observed in 112 (78.3%). of these, 103 (92%) were Gram-negative bacteria and 9 (8%) were Gram-positive bacteria (Table 7).

**Table 7:** Multidrug resistance profile of bacteria isolated from blood culture of newborns suspected of sepsis at Hawassa University Comprehensive Specialized Hospital from February 2021 to November 2021.

Bacterial isolates	Resistance patterns						
	R0	R1	R2	R3	R4	≥ R5	MDR n (%)
*Klebsiella* species (61)	–	–	3	32	21	5	58 (95)
*Enterobacter* species (4)	–	–	1	2	1	–	3 (75)
*Pseudomonas* species (11)	–	–	–	1	3	7	11 (100)
*Escherichia coli* (7)			1	2	1	3	6 (85.7)
*Acinetobacter* species (16)	–	–	–	2	5	9	16 (100)
*Enterobacter aglemerans* (6)	–	–	–	4	1	1	6 (100)
*Serratia marcescens*(2)	–	–	–	–	2	–	2 (100)
*Citrobacter* species (1)	–	–	–	–	–	1	1 (100)
*Enterococcus* species (26)	–	18	–	8	–	–	8 (30.8)
*Staphylococcus aureus* (9)	–	6	2	1	–	–	1 (11.1)

*NICU* Neonatal intensive care unit, *HUCSH* Hawassa comprehensive specialized hospital, *MDR* Multiple drug resistance (resistance to three or more drugs categories), *R0* No resistance at all, *R1* resistant to one antimicrobial class, *R2* resistant to two antimicrobials class, *R3* Resistant to three antimicrobials class, *R4* resistant to four antimicrobials class, *R5* resistant to five or more antimicrobials class.

## Discussion

The etiology of neonatal sepsis is heterogeneous; it varies from country to country; for instance, *S. agalactiae* was among the leading cause of neonatal sepsis in the United States until Intrapartum Antibiotic Prophylaxis, a prevention strategy, was implemented^12^. However, this bacterium is rarely isolated from newborns suspected of sepsis in Ethiopia^4,11^. The prevalence of neonatal sepsis in the present study is 36.5% of which 53.1% were early-onset and 46.9% were late-onset sepsis. The finding is in agreement with studies conducted in different parts of Ethiopia and other countries (30–36%)^4,11–17^. In contrast to our study, lower than 30% prevalence of neonatal sepsis was reported in Southeast Ethiopia^3^, India^18^; and less than 20% prevalence was reported in Pakistan^13^, Ghana^19^, Uganda^20^, Kenya^21^, and Nepal^22^. We have noted that even higher prevalence of neonatal sepsis in Shashamane, Ethiopia (77.6%)^23^ and Gondar, Ethiopia (64.8%)^23^. The variation in prevalence might be due to prior antimicrobial administration before sample collection, and methods used to confirm neonatal sepsis. Some studies^23,24^ did not involve culture; their report depends only on clinical findings only.

In the present study, 75.5% of isolates were Gram-negative bacteria while Gram-positive bacteria accounted only for 24.5%. The results are consistent with another study conducted in Bhutan^25^. Gram-negative bacteria were the most commonly isolated bacteria causing neonatal sepsis in this study, which is in line with studies conducted by Eshetu et al.^10^ and Lamba et al.^8^. This study revealed that most EOS (86.8%) are caused by Gram-negative bacteria. Among Gram-positive bacteria, *Enterococcus* species were the most prevalent; this bacterium predominates in late-onset sepsis indicating horizontal transmission. We did not come across other studies that correlate with our findings^11^. The high prevalence may be related to fecal contamination of newborns during birth. Even though Ethiopia is not implementing the Intrapartum Antibiotic prophylaxis strategy recommended for Group B *Streptococci*, we did not isolate Group B *Streptococcus* from newborns suspected of sepsis.

In the current study, the most predominant bacteria were *Klebsiella* species while the least recovered bacteria were *Citrobacter* species. Similarly, *Klebsiella* species were among the most common pathogens reported in bloodstream infections^11^. *Klebsiella* species (47.4%) was the predominant isolate from EOS cases. Similar finding was reported by Lamba et al.^8^. Early-onset sepsis is usually acquired from the mother during or before birth through vertical transmission; this transmission is amenable for prevention as learned from group B *Streptococci*. The source of late-onset sepsis is mostly unknown; it can be transferred to newborns from mothers, delivery attendants, and family members after birth. In this study, we have observed high numbers of *Klebsiella* species from newborns with early-onset and late-onset sepsis.

*Klebsiella* species can reside in the hospital environment, gastrointestinal tract, birth canal, and on the surfaces of medical devices^26^. These species is an important public health concern especially in health care settings with only a few antimicrobial agents to treat^27^. In this study, newborns infected with *Klebsiella* species might have acquired the bacterium vertically from the mother before birth (early-onset sepsis) or horizontally (late-onset sepsis). A study from Hawassa indicated that 3.2% vaginal colonization rate of *Klebsiella* species among pregnant women in the third trimester^28^. This indicates the possibility of vaginal colonization as a pre-requisite for early-onset sepsis similar to Group B *Streptococci*. However, in our study, a high proportion of early-onset sepsis due to *Klebsiella* species may not be explained by just vaginal colonization. In addition, a study by Bitew et al. isolated a high proportion of *Klebsiella* species from medical devices and the hospital environment of the NICU of HUCSH^29^ indicating the possible source of infection. To confirm the source of infection: vertical or horizontal, molecular characterization may be needed.

The odd of developing sepsis in newborns from mothers with premature rupture of the membrane is about 12 times as compared to their counterparts. This finding is similar to studies conducted in Ethiopia^6^ and Kenya^21^. Premature rupture of the membrane may facilitate the proliferation of bacteria that are found in the birth canal and are able to ascend to the amniotic sac and initiate infection. This study showed that newborns that were born via cesarean section were less likely to develop sepsis which is consistent with studies conducted in Ghana^5,30^. This might be due to the fact that newborns delivered through cesarean section are not exposed to bacteria that reside in the recto-vaginal compartment of pregnant women.

This study indicated that females are about 2 times more likely to develop sepsis as compared to male newborns. Unlike our study, a report from Gondar indicated that male newborns are about 4 times more likely to develop sepsis^24^. Reasons for admission to NICU such as low birth weight showed a significant association with neonatal sepsis [AOR = 3.17 (95% CI 1.278–7.859)]. This finding is in line with studies conducted in Ethiopia^2,6,11^ and Mexico^31^. The possible reason might be, the underweight newborns are more likely to possess immature immunity and are vulnerable to infection^32^. Newborns without respiratory support were about 3 times at risk of developing sepsis as compared to those who had nasal prongs. A similar finding was reported in China^17^. This could be due to newborns who were not on respiratory support were easily exposed to microbes dispersed in the surrounding environment or objects.

Overall, 10.2% to 99.1% of Gram-negative bacteria were resistant to tested antibiotics. Most Gram-negative bacterial isolates were susceptible to meropenem, ciprofloxacin, and amikacin. This is in agreement with the antibiotic resistance profile reported in India^8^, Ethiopia^11^, and Pakistan^13^. In addition, most bacteria were susceptible to ciprofloxacin which is consistent with findings reported in Sikkim^18^. A relatively high proportion of Gram-positive bacterial isolates showed susceptibility to ciprofloxacin, erythromycin, and chloramphenicol. On the other hand, a high proportion of Gram-positive bacteria was resistant to penicillin (82.9%), ampicillin (80%), and trimethoprim-Sulfamethoxazole (55.6%). All *S. aureus* were susceptible to linezolid whereas 55.6% were resistant to penicillin. In contrast to this, 94.7% of *S. aureus* isolated from the nasal cavity of hospitalized patients were resistant to penicillin^33^. 30.7% and 92.3% of *Enterococcus* species were resistant to vancomycin and penicillin respectively. For *Enterococcus* species that are resistant to vancomycin, there are few options to treat sepsis caused by them.

78.3% of bacteria isolated in the current study were multi-drug resistant where the majority of it occurs among Gram-negative bacteria (92%). The finding is in agreement with studies conducted in different parts of Ethiopia^10,31^. All *Pseudomonas* species*, Acinetobacter* species, *Enterobacter aglemerans* were MDR. This is alarming as we are left with few antibiotics to treat infections caused by these organisms.

### Limitations of the study

Since we have used a convenient non-probability sampling technique, there might be the introduction of selection bias. We did not attempt to isolate anaerobic bacteria because of a lack of facility.

## Conclusions

The overall prevalence of culture-confirmed neonatal sepsis was 36.5%. Gram-negative bacteria were the dominant pathogens causing neonatal sepsis among newborns. *Klebsiella* species was the most common bacteria followed by Gram-negative non-lactose fermenting bacteria. Most Gram-negative bacteria are resistant to multiple antibiotics. Ciprofloxacin was the most effective drug for Gram-positive and Gram-negative bacteria. Gestational age, respiratory support, and ways of delivery were identified factors that significantly increase the risk of neonatal sepsis.

## Data Availability

The datasets generated and/or analysed during the current study are not publicly available due the regulation of Hawassa University but are available from the corresponding author on reasonable request.

## Abbreviations

EOS: Early onset sepsis
LOS: Late onset sepsis
HUCSH: Hawassa university comprehensive specialized hospital
NICU: Neonatal intensive care unit
